# Mobility and sales activity during the Corona crisis: daily indicators for Switzerland

**DOI:** 10.1186/s41937-020-00055-9

**Published:** 2020-08-24

**Authors:** Florian Eckert, Heiner Mikosch

**Affiliations:** KOF Swiss Economic Institute, ETH Zurich, Leonhardstrasse 21 LEE, Zurich, 8092 Switzerland

**Keywords:** Corona crisis, Mobility, Sales activity, Daily indicators, C38, D12, E32, H12, I12

## Abstract

This paper documents daily compound indicators on physical mobility and sales activity in Switzerland during the Corona crisis. We report several insights from these indicators: The Swiss population substantially reduced its activities already before the shops closed and before the authorities introduced containment policies in mid-March 2020. Activity started to gradually recover from the beginning of April onwards, again substantially before the first phase of the shutdown easing started at the end of April. Low physical mobility during the second half of March and during April likely contributed to the quick fall in new COVID-19 infections since mid-March. The sharp drop in economic activity in consumer-related services during March and April and the gradual recovery in these sectors since May correlate strongly with the reduction and subsequent gradual resurgence of mobility. In addition, while activity within Switzerland was back to normal levels by late June, activity of Swiss residents outside of Switzerland was still below normal.

## Introduction

The Corona pandemic caused a sharp drop in Swiss economic activity in March and April 2020^1^. The recovery since the end of April has progressed at a very high pace compared to normal recoveries. Most macroeconomic indicators, such as business tendency surveys or consumer sentiment indices, failed to keep track of the sudden down and up, since they are sampled at a monthly frequency and published with delay. As a consequence, there was a need to collect and publish new daily or weekly data series, such as credit card transactions or traffic volumes, with the aim to monitor economic and social activity closer to real time. However, a challenge is that these higher frequency series often cover only specific aspects of economic or social activity. In addition, most of the series fluctuate strongly, move partly idiosyncratically and often have (multiple) strong seasonal patterns. It is thus very difficult to infer the state of economic or social activity from the real-time inspection of the individual noisy series.

To overcome these challenges, shortly after the start of the Corona crisis, we started collecting a diverse set of daily and weekly data series. We eliminated seasonal patterns, dealt with missing data and ragged edges, extracted common cyclical components via principal component analysis and applied different scaling methods. From this procedure, we obtained daily compound indicators on physical mobility, on sales activity, on economic activity inside Switzerland and on international travel activity of Swiss residents. Also, we constructed an encompassing activity indicator. Since March, we publish the indicators in the KOF High Frequency Economic Monitoring Dashboard (2020) for download and visual inspection. The compound indicators are easily interpretable and nicely track the sudden down and up of activity during the crisis^2^.

Our focus on mobility and spending activity of the Swiss population has two reasons. First, social distancing is a key factor to contain the spread of the virus. Physical mobility is a proxy for social distancing, since the latter is largely achieved by a reduction in the former. Thus, by providing an indicator on the degree of physical (im)mobility, we intended to help monitoring the degree of social distancing exerted by the Swiss population during the crisis. Second, there was an increasing tendency of the Swiss people to reduce their activities well before the strict imposition of physical distancing rules. This response to the spread of the virus was certain to induce a steep fall in economic activity in consumer-related services. We wanted to help tracking this development by providing a compound indicator on spending activity in Switzerland. Indeed, our compound indicators have been cited by Swiss sources in order to report on mobility, sales activity and economic activity in general during the Corona crisis^3^.

This paper documents the construction procedure of the daily compound indicators. In addition, the paper provides several insights on mobility and sales activity in Switzerland during the Corona crisis. These insights are summarized in the conclusion of the paper.

The paper is structured as follows. Section 2 documents the high-frequency time series underlying the compound indicators. It also describes how we deal with seasonality patterns, missing values and ragged edges. Section 3 describes the construction of the indicators and discusses the possible scaling methods that allow for a meaningful interpretation of the indicators. Section 4 briefly reviews the progression of the COVID-19 pandemic in Switzerland. Section 5 then presents several insights on mobility and sales activity in Switzerland during the crisis. Section 6 concludes.

## Data

The series underlying the indicators cover aspects of physical mobility and/or economic activity related to purchases and sales of goods and services. The majority of the series cover Switzerland as a whole, whereas some series are limited to the Zurich region. We take these latter series as the best available proxies for overall Switzerland. The data come from various sources and are assembled and provided by the Statistical Office of the Canton of Zurich as part of its Corona crisis monitoring initiative (Gesellschaftsmonitoring COVID19 (2020)). Further series are assembled and provided by the KOF High Frequency Economic Monitoring Dashboard (2020). The COVID-19 infection numbers used in Section 5 are collected by the Swiss cantons and are also provided by Gesellschaftsmonitoring COVID19 (2020)^4^. A comprehensive overview of the data can be found in Appendix A.1.

Most time series used in this paper are observed at daily frequency and start on January 7, 2020. Some series come at weekly frequency and start in the second week of January 2020. We temporarily disaggregate these series to daily frequency by linear interpolation, assuming the actual observation to take place in the middle of the week. The inclusion of the weekly variables provides additional information on the broad trajectory of the principal component. This benefit outweighs the potential drawback of less precision at the highest frequency. The tradeoff between information and precision is further investigated in Appendix A.2. Any missing observations for the daily series are equally filled using linear interpolation.

Most of the daily series have strong weekly seasonality patterns. We adjust for the weekly seasonality using the seasonal-trend decomposition (STL) based on locally estimated scatterplot smoothing (LOESS) (Cleveland et al. 1990). Some series are likely to have very little annual seasonality, while others such as pedestrian traffic are usually subject to strong annual seasonal patterns. Due to the limited data availability before January 2020, it is not possible to adjust the series for annual seasonality. However, as long as there is sufficient variation in the scale of annual seasonality between the series, these commonalities are likely to be picked up by components other than the first principal component based on which we construct the compound indicators (see Section 3). Some series are published with a delay of 1 to 3 days. To fill the missing values at the current edge, we project these series using the trend component of the STL method^5^.

## Indicator construction

In order to construct the compound indicators from the underlying series, we compute the first principal component using a singular value decomposition of the series. All series are centred at zero and are scaled to have unit variance before the principal component analysis (PCA) is applied. To check for robustness, we also experimented with various forms of factor analysis (e.g., Stock and Watson (2011)) and found the indicators to behave very similar as compared to PCA.

For the purpose of standardization, we index the average of the period from January 7, 2020 to February 28, 2020 to 100 for each resulting indicator. Underlying this standardization is the presumption that the levels of the series have been fluctuating in a normal range during the aforementioned period, which was prior to the outbreak of the crisis in Switzerland. We further standardize the indicators by setting the individual minimum value of each indicator to zero. We call this standardization the “minimum-maximum standardization”. The standardization procedure acknowledges the fact that, due to the shortness of the indicators, one should be careful in making statements on how the indicators compare to each other in terms of the size of their decrease during the crisis. Nevertheless, the standardization allows for statements on the relative position of the indicators with respect to their pre-Corona level (= 100) and their minimum level (= 0) during the peak of the crisis^6^.

We compile five compound indicators. First, the *mobility indicator* includes series related to physical mobility, such as the frequency of persons passing at important train stations, the frequency of private transport vehicles (cars etc.) counted at important measuring stations and the daily average geographical distance covered by a representative sample of the Swiss population. Table 1 in Appendix A.1 provides the full list of variables included in this indicator. Importantly, the mobility indicator can be seen as a proxy for the degree of social distancing exerted by the Swiss population, since the latter is largely achieved by a reduction in the former. Second, the *sales activity indicator* encompasses series that are related to the purchase and sales of goods and services such as the volume and number of credit and debit card transactions or Google searches for shopping goods. The full variable list is given in Table 2 in Appendix A.1. Third and fourth, the *indicator on activity inside Switzerland* comprises all available series that reflect economic activity inside Switzerland, whereas the *indicator on international travel activity of Swiss residents* includes series such as Swiss credit card transactions abroad and the number of flight arrivals/departures at Zurich Airport. Tables 1 and 2 list all variables included in the aforementionend indicators. Finally, the *encompassing actvity indicator* comprises all available high-frequency series and, thereby, attempts to capture the daily activity of the Swiss population as broadly as possible given the available data.

## Corona chronology

The first recorded Swiss COVID-19 case occurred in the canton of Ticino and was confirmed on February 25 by the Swiss Federal Office of Public Health (FOPH). On February 28, the Swiss government declared the situation in Switzerland a “special situation” according to Swiss pandemic law and banned events with more than 1000 participants. On March 1, the FOPH launched the prevention campaign “How we protect ourselves” including public posters, leaflets, a telephone hotline and a website. The campaign included recommendations on personal hygiene and social distancing measures.

The virus quickly started to spread throughout Switzerland with 29 recorded cases by March 1, 125 recorded cases by March 5 and 542 recorded cases by March 10. The first recorded Swiss COVID-19 fatality occurred on March 5.

On March 16, the Swiss government declared an “extraordinary situation” according to Swiss pandemic law. All shops (except food stores and pharmacies), markets, restaurants, bars, entertainment and recreation facilities had to be closed (“lockdown”). Private and public events were prohibited. Cross-border mobility for non-resident foreigners was heavily restricted. Further, companies started to introduce home office as a temporary working standard. On March 20, the government banned gatherings of more than five people. In its prevention campaign, the FOPH now appealed to everyone to generally stay at home. The slogan “Stay at home” was disseminated by virtually all Swiss media following the recommendations of the FOPH and the Swiss Media Association.

The reopening of the economy and social life took place in several stages: From April 27 onwards, selected shops, e.g., hairdressers, do-it-yourself stores and garden centres, were allowed to reopen, provided that they introduced certain social distancing and health measures (“Easing Phase 1”). Shops, restaurants, markets, museums and libraries could reopen on May 11, again provided the implementation of certain social distancing and health measures. Also, compulsory schools reopened (“Easing Phase 2”). From June 6 onwards, private and public events up to 300 persons were again permitted, and secondary schools opened. Further, leisure and tourism businesses, such as mountain railways, camping sites, casinos, amusement parks, zoos, botanical gardens and swimming pools, were allowed to reopen (“Easing Phase 3”). On June 15, Switzerland completely opened its borders to all EU/EFTA states. Cross-country shopping tourism, which is important for border regions, was also permitted again.

## Results

**Fig. 1:**
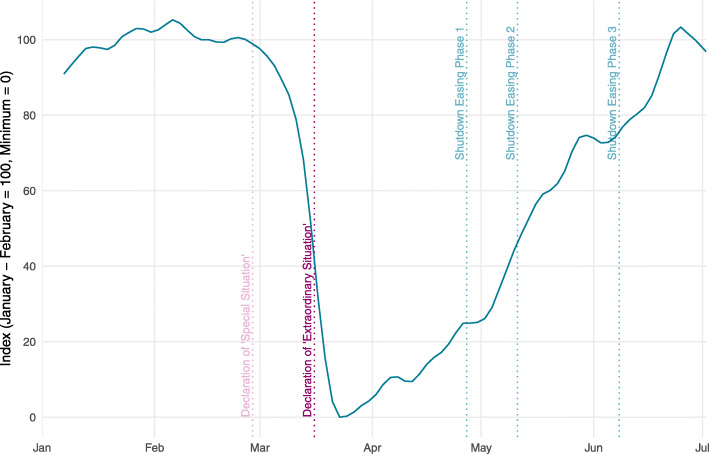
Activity indicator for Switzerland

Figure 1 shows the encompassing activity indicator in its minimum-maximum standardization version. According to the indicator, activity in Switzerland was at a normal level by the end of February and then quickly started to go down. On March 15, activity was at about half its pre-Corona level in relation to the overall minimum level, which was reached on March 23. An important insight here is that, in response to the perceived infection risk, Swiss people had substantially reduced their activities already before the shop closings, the general conversion to home office and the appeal to generally stay at home in mid-March.

From the end of March onwards, activity gradually started to recover. When the government introduced the first stage of relaxations on April 27, activity was already back to around one fourth between its minimum level and its pre-Corona level. One explanation is that the Swiss population increased its activity as a reaction to the strong decrease in the daily COVID-19 infections (see below). An additional factor might be that the Swiss government had announced on April 16 a timetable for relaxing the containment measures. Activity started to increase quickly after this announcement.

The pace of normalization increased further since early May. The temporary flattening of the indicator at the end of May and the beginning of June may be attributed to poor weather. Since then, normalization has continued with the activity being back to pre-Corona levels in the last week of June.

**Fig. 2:**
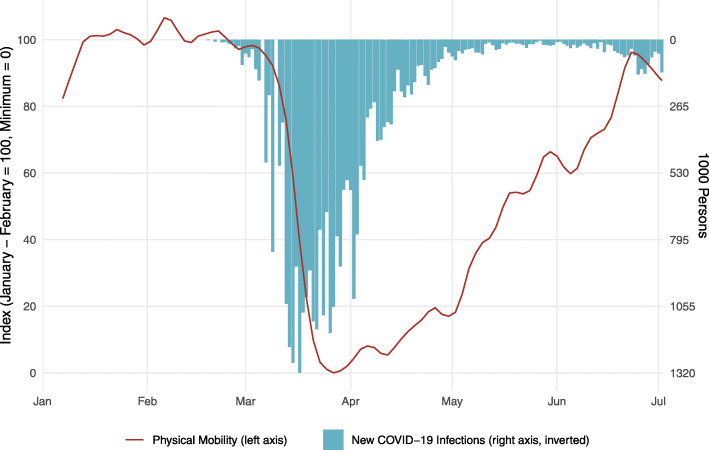
Mobility and evolution of the pandemic in Switzerland

Figure 2 shows the daily numbers of new COVID-19 infections jointly with the indicator on physical mobility, which serves as a proxy for the degree of social distancing exerted by the Swiss population. We construct the daily new infection numbers by leading the officially recorded infection numbers by 6 days, since the incubation period for COVID-19 is 5–6 days on average. As can be seen from the figure, daily new infection numbers strongly increased from early until mid-March, when mobility was still at half the level between its pre-Corona level and its minimum level. The pattern suggests that the social distancing behaviour (proxied by immobility) of the Swiss population during the second half of March and during April has contributed to the quick fall in new infection cases.

**Fig. 3:**
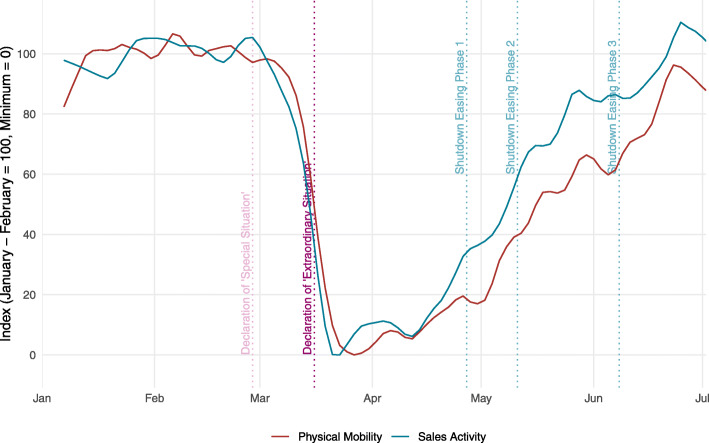
Physical mobility and sales activity

Figure 3 displays the indicator on physical mobility together with the indicator of sales activity. The figure shows that, during the Corona crisis, physical mobility and spending activity moved very closely together, despite the fact that the two indicators actually cover quite different series. Both physical mobility and shopping activity decreased for three consecutive weeks from the beginning of March onwards. Since April, both indicators have been steadily trending upwards again, and by the last week of June, they returned to their pre-Corona levels. The joint pattern of the indicators suggests that the decisions of the Swiss people to reduce their physical mobility in response to the rise in infection numbers has contributed to the steep fall in economic activity during March and April. This affected particularly consumer-related services such as wholesale and retail trade, transportation and logistics, and hotels and restaurants. Conversely, the pattern suggests that the gradual recovery in these industries since May is related to the resurgence of mobility.

**Fig. 4:**
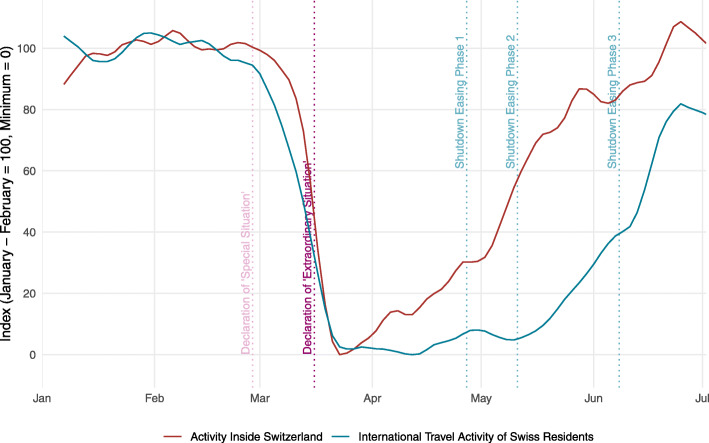
Activity inside Switzerland and international travel activity

Figure 4 displays the indicator on activity inside Switzerland and the indicator on international travel activity of Swiss residents. Activity inside Switzerland started to recover from the end of March onwards and was back to its pre-Corona level by the last week of June. In contrast, activity of Swiss residents travelling abroad started to recover in mid-May only. By the end of June, it was still below its levels before the crisis. This pattern conforms to the fact that, just as other governments worldwide, the Swiss authorities have been more reluctant to relax restrictions for travelling abroad than for activities inside the country.

## Conclusion

Shortly after the start of the Corona crisis in Switzerland, we began to publish daily compound indicators on physical mobility and spending activity of the Swiss population. Daily updates were made publically available via the KOF High Frequency Economic Monitoring Dashboard (2020). Thereby, we intended to help the Swiss public in monitoring its own behaviour during the crisis, where this self-monitoring is a key ingredient to successfully overcome the crisis.

**Table 1:** Data overview

Series	Frequency	Coverage	Which indicator	Source
Number of flight arrivals/departures at Zurich Airport	Daily	–	Encompassing, physical mobility, Swiss travelling abroad	Zurich Airport
Number of train arrivals/departures at Zurich Main Station	Daily	ZH & CH	Encompassing, physical mobility, inside Switzerland	Swiss Federal Railways (SBB CFF FFS)
Frequency of persons passing at train station Zurich Hardbrücke	Daily	ZH	Encompassing, physical mobility, inside Switzerland	Swiss Federal Railways (SBB CFF FFS)
Private transport frequency, important measuring stations	Daily	ZH	Encompassing, physical mobility, inside Switzerland	City of Zurich
Pedestrian traffic frequency, important measuring stations	Daily	ZH	Encompassing, physical mobility, inside Switzerland	City of Zurich
Median day distance, all ages, representative population sample	Daily	CH	Encompassing, physical mobility, inside Switzerland	Intervista
Median day distance, 15–29 years, representative population sample	Daily	CH	Encompassing, physical mobility, inside Switzerland	Intervista
Median day distance, 30–64 years, representative population sample	Daily	CH	Encompassing, physical mobility, inside Switzerland	Intervista
Median day distance, 65–79 years, representative population sample	Daily	CH	Encompassing, physical mobility, inside Switzerland	Intervista
Median day distance, men, representative population sample	Daily	CH	Encompassing, physical mobility, inside Switzerland	Intervista
Median day distance, women, representative population sample	Daily	CH	Encompassing, physical mobility, inside Switzerland	Intervista
Median day distance, urban, representative population sample	Daily	CH	encompassing, physical mobility, inside Switzerland	Intervista
Median day distance, rural, representative population sample	Daily	CH	Encompassing, physical mobility, inside Switzerland	Intervista
Usage online learning platform Mindsteps (home schooling)	Daily	Swiss German CH	Encompassing, physical mobility, inside Switzerland	University of Zurich
Usage online math training platform (home schooling)	Daily	CH	Encompassing, physical mobility, inside Switzerland	LMVZ Lehrmittelverlag Zürich
Google searches mobility (ground transportation), frequency	Daily	CH	Encompassing	trenEcon
Google searches travel abroad (flight/holiday bookings), frequ.	Daily	CH	Encompassing, travelling abroad	trenEcon

*Abbreviations*: *CH* Switzerland, *ZH* Zurich

**Table 2:** Data overview continued

Series	Frequency	Coverage	Which indicator	Source
Non-online retail sales, volume in CHF	Daily	CH	Encompassing, sales activity, inside Switzerland	SIX BBS AG
Non-online retail sales, number debit card transactions	Daily	CH	Encompassing, sales activity, inside Switzerland	SIX BBS AG
ATM cash withdrawal, volume in CHF	Daily	CH	Encompassing, sales activity, inside Switzerland	SIX BBS AG
ATM cash withdrawal, frequency	Daily	CH	Encompassing, sales activity, inside Switzerland	SIX BBS AG
Swiss debit card transactions abroad, volume in CHF	Daily	Abroad	Encompassing, sales activity, Swiss travelling abroad	SIX BBS AG
Swiss debit card transactions abroad, frequency	Daily	Abroad	Encompassing, sales activity, Swiss travelling abroad	SIX BBS AG
Swiss credit card transactions, volume in CHF	Weekly	CH	Encompassing, sales activity, inside Switzerland	Swiss Payment Association
Swiss credit card transactions, frequency	Weekly	CH	Encompassing, sales activity, inside Switzerland	Swiss Payment Association
Swiss credit card transactions, on site, volume in CHF	Weekly	CH	Encompassing, sales activity, inside Switzerland	Swiss Payment Association
Swiss credit card transactions, remote/online, volume in CHF	Weekly	CH	Encompassing, sales activity, inside Switzerland	Swiss Payment Association
Swiss credit card transactions, on site, frequency	Weekly	CH	Encompassing, sales activity, inside Switzerland	Swiss Payment Association
Swiss credit card transactions, remote/online, frequency	Weekly	CH	Encompassing, sales activity, inside Switzerland	Swiss Payment Association
Swiss credit card transactions, abroad, volume in CHF	Weekly	Abroad	Encompassing, sales activity, Swiss travelling abroad	Swiss Payment Association
Swiss credit card transactions, abroad, frequency	Weekly	Abroad	Encompassing, sales activity, Swiss travelling abroad	Swiss Payment Association
Google searches watches and jewellery, frequency	Daily	CH	Encompassing, sales activity	trendEcon
Google searches clothing and shoes, frequency	Daily	CH	Encompassing, sales activity	trenEcon
Google searches gardening and home improvement, frequency	Daily	CH	Encompassing, sales activity	trenEcon

*Abbreviations*: *CH* Switzerland, *ZH* Zurich, *CHF* Swiss Francs

This paper presented compound indicators on physical mobility, sales activity, activity inside Switzerland and international travel activity of Swiss residents, as well as an encompassing activity indicator. The indicators have been constructed from a set of time series including the frequency of persons passing at important train stations, the frequency of private transport vehicles (cars etc.) counted at important measuring stations, flight departures and arrivals, the volume and number of credit and debit card transactions and Google searches for shopping goods, amongst others.

We gained several insights from the daily indicators. To begin with, the Swiss population had substantially reduced its activities already before the shop closings, the general conversion to home office and the appeal to generally stay at home in mid-March 2020. This behaviour may be a response to the perceived infection risk. Next, from the beginning of April onwards, activity gradually recovered. When the Swiss government introduced the first phase of relaxations on April 27, activity was already back to one fourth of its pre-Corona level relative to its minimum level. Also, a comparison of physical mobility and COVID-19 infection numbers suggests that social distancing behaviour (proxied by immobility) of the Swiss population during the second half of March and during April correlates strongly with the quick fall in new infection cases since mid-March. Further, physical mobility and spending activity moved very closely together during the Corona crisis. The joint pattern suggests that the decisions of the Swiss people to reduce their mobility has contributed to the steep fall during March and April in value added of the consumer-related service industries. The pattern further suggests that the gradual recovery in these industries since May is related to the gradual resurgence of mobility. Finally, while the activity inside Switzerland was back to normal levels by the end of June, the activity of Swiss residents travelling abroad was still below normal.

Physical distancing has proven to be effective in suppressing the Corona pandemic until June 2020. In order to contain a second virus wave, precautionary measures will stay in place for the foreseeable future. While it is too early to determine the level of social distancing that keeps the pandemic in check, it seems certain that this level lies above pre-Corona levels. The Swiss population decreased its mobility already well before the lockdown, and it has increased its activity steadily instead of abruptly during the easing steps. This behaviour may be interpreted as a sign for the individual responsibility of the Swiss people. As a consequence, timely information from the authorities on new infections seems to be important to contain new virus waves. Timely and effective dissemination of new insights on how individuals should adapt their personal behaviour in order to reduce the infection risk seems to be another key ingredient to overcome the crisis.

## Appendix

### A.1 Data description

The following tables provide an overview of the data used in this paper, along with meta information such as frequency, coverage and source. The tables furthermore show which series contribute to which compound indicator.

### A.2 Robustness checks

**Fig. 5:**
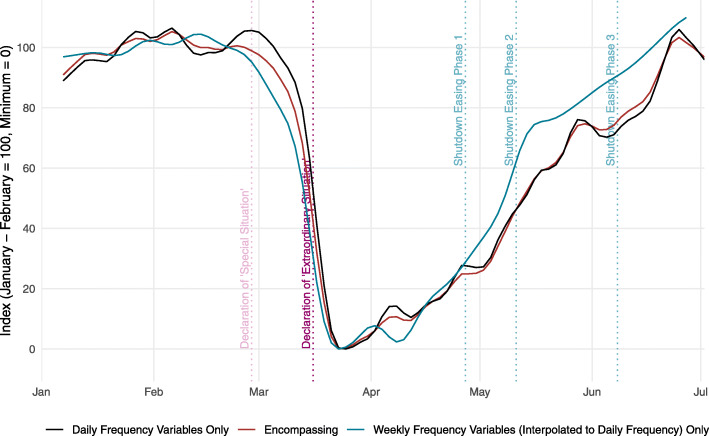
Activity indicator excluding either weekly or daily variables

Figure 5 shows the encompassing activity indicator for Switzerland together with two subindicators including either only the daily frequency variables or only the weekly frequency variables, which have been interpolated to daily frequency as described in Section 2. The encompassing activity indicator and the subindicator including the daily series only move closely together. While the interpolated weekly series are smoother than the daily series due to the interpolation involved in the temporal disaggregation, the resulting indicator is only slightly less volatile than the indicator including the daily data only. The impact of interpolated weekly series in the data set is therefore likely to be small. Differences between the two indicators arise due to the nature of the underlying series. The weekly series reflect only credit card transactions, while the daily series cover a broad set of indicators.

### A.3 Alternative scaling

**Fig. 6:**
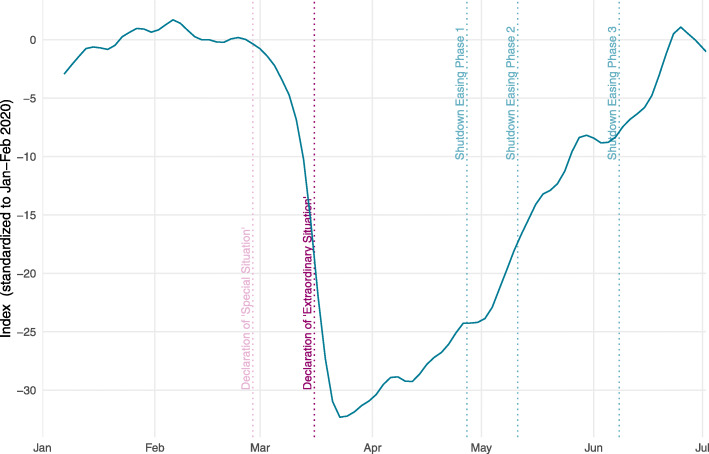
Activity indicator for Switzerland (mean-variance standardization)

Figure 6 presents the encompassing activity indicator for Switzerland in its mean-variance standardization version. On March 23, activity has been, in terms of Jan.–Feb. 2020 standard deviations, more than 30 standard deviations below its pre-Corona level. This reveals how extreme the drop in activity was due to the pandemic.

Scaling using mean-variance standardization hinges on the assumption that an indicator’s standard deviation of the period Jan.-Feb. 2020 is representative for its standard deviation during “normal times.” As far as this assumption is fulfilled for each indicator, they can be readily compared to each other in terms of the size of their decrease during the crisis.

**Fig. 7:**
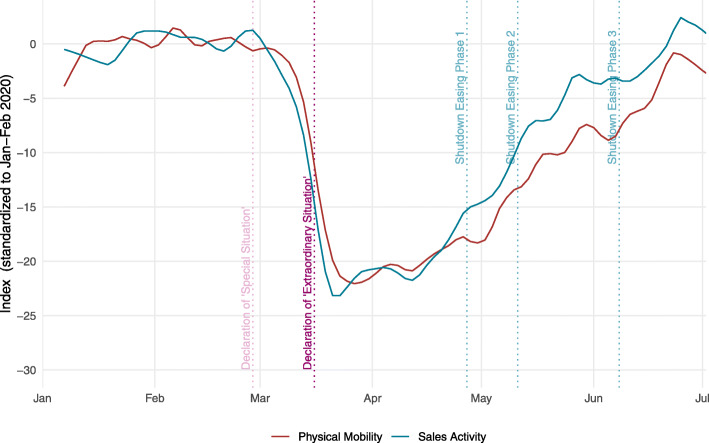
Physical mobility and sales activity (mean-variance standardization)

Figure 7 displays the indicator on physical mobility together with the indicator of sales activity both in their mean-variance standardization version. Both physical mobility and shopping activity fell by somewhat more than 20 standard deviations below their respective pre-Corona levels by the third week of March 2020 (in terms of Jan.–Feb. 2020 standard deviations). The equally strong drop of the indicators confirms their close relation in addition to the results presented in Section 5.

## Data Availability

The data used for this paper are openly available at Gesellschaftsmonitoring COVID19 (2020) and KOF High Frequency Economic Monitoring Dashboard (2020).

## Footnotes

1 See, e.g., Bachmann et al. (2020).

2 Going beyond our aggregate indicator on sales activity for Switzerland, Monitoring Consumption Switzerland (2020) uses detailed disaggregate payment data to shed light on consumer spending in Switzerland and how it is impacted by the Corona crisis. To highlight two other recent projects, Burri and Kaufmann (2020) develop a “fever curve” for the Swiss economy using daily financial market and news data. The trendEcon (2020) project provides daily Google search-based economic indicators on the perceived economic situation in Switzerland and on several consumption good categories.

3 See, e.g., *Neue Zürcher Zeitung*, May 18, 2020, p. 15, *Tagesanzeiger*, May 26, 2020, *Republik*, June 8, 2020, *Neue Zürcher Zeitung*, June 17, 2020, p. 18 and *Volkswirtschaftsdirektion des Kantons Zürich: Zürcher Wirtschaftsmonitoring*, Juni 2020 (*Economics Directorate of the Canton of Zurich: Economic Monitoring*, June 2020).

4 www.corona-data.chassembles and visualizes statistics related to Swiss COVID-19 cases and fatalities from official sources. See Guidotti and Ardia (2020) for cross-country data related to COVID-19.

5 The series on flight arrivals and departures at Zurich airport starts on January 17, 2020. We backcast the series using again the trend component of the STL method.

6 For an alternative standardization, we index each indicator to an average of zero over the period from January 7, 2020 to February 28, 2020. Then, we divide each observation by the standard deviation of the aforementioned period. As a consequence, the indicator units tell us how much each indicator is currently below (or above) its pre-Corona level in terms of Jan.–Feb. 2020 standard deviations. Selected figures using this alternative representation can be found in Appendix A.3.
